# Robustaflavone induces G0/G1 cell cycle arrest and apoptosis in human umbilical vein endothelial cells and exhibits anti-angiogenic effects in vivo

**DOI:** 10.1038/s41598-020-67993-5

**Published:** 2020-07-06

**Authors:** Woo Kyung Sim, Jong-Hwa Park, Ki-Young Kim, In Sik Chung

**Affiliations:** Department of Genetic Engineering and Graduate School of Biotechnology, Kyung Hee University, Yongin, 17104 Republic of Korea

**Keywords:** Chemotherapy, Pharmaceutics

## Abstract

We investigated the anti-angiogenic and pro-apoptotic effects of robustaflavone (RF), a naturally occurring biflavonoid, on human umbilical vein endothelial cells (HUVECs). RF inhibited HUVEC proliferation and showed cytotoxicity that inhibited HUVEC viability. RF-induced apoptosis was characterized by flow cytometry and caspase 3 analysis. We found that RF increased the number of sub-G1 cells and terminal deoxynucleotidyl transferase dUTP nick end-labeled cells. Additionally, RF induced caspase 3 and poly (ADP-ribose) polymerase activation. Potential molecular targets were identified using a human apoptosis antibody array. RF upregulated Bax, Bad, cleaved caspase 3, p21, and phosphorylated p53 levels. RF induced mitochondrial membrane potential loss and the release of cytochrome c and apoptosis-inducing factor. Cell cycle arrest at G0/G1 phase and the downregulation of Cdk4, Cdk6, and cyclin D1 expression were induced by RF. In vivo anti-angiogenic effects were investigated using a tumor allograft animal model and a Matrigel plug assay. RF reduced the volumes and weights of CT-26 cell-derived tumors. The blood vessel density was significantly decreased in RF-treated tumors. RF also inhibited VEGF-A-stimulated blood vessel formation in vivo in Matrigel plugs. These results suggest that RF can potentially inhibit angiogenesis-dependent tumor growth and metastasis.

## Introduction

Angiogenesis involves the formation of new bloods from pre-existing blood vessels and plays essential roles in development, wound healing, ovulation, inflammation, and tumor growth^1^. Angiogenesis is a key process for tumor growth and the development of metastatic tumors because it supplies nutrients and oxygen to tumors^2^. Therefore, angiogenesis is a major target for new cancer chemotherapeutics. Anti-angiogenic therapy is considered an effective anti-cancer strategy^3,4^. Several anti-angiogenic agents have been studied preclinically and clinically^4^. However, some of these anti-angiogenic agents can cause endothelial dysfunction and a decrease in the number of blood vessels in normal tissues^5^. Resistance to anti-angiogenic agents can reduce the efficiency of anti-angiogenic therapy^6^. Therefore, it is necessary to develop safer and more effective anti-angiogenic agents.

Robustaflavone (RF) is a natural biflavonoid compound composed of two molecules of apigenin (5,7,4′-trihydroxyflavone) joined via a biaryl linkage between the 6-position of one molecule and the 3′-position of the other. RF has been isolated from several plants including *Rhus succedanea*, *Garcinia multiflora*, *Ochna schweinfurthiana*, *Dietes bicolor*, and *Selaginella* species^7–10^. It possesses anti-viral, anti-allergic, anti-inflammatory, and anti-tumor activities^8–12^. RF inhibits the replication of hepatitis B virus and has a strong inhibitory effect against influenza A and influenza B viruses^11,12^. It exerts anti-allergic effects by inhibiting antigen-induced β-hexosaminidase and anti-inflammatory activity by blocking peroxide anion generation^10^. In addition, it is a major anti-cancer compound present in the ethyl acetate extract of *Selaginella* species^8^. RF may exhibit anti-tumor activity and reduce the viability of hepatocellular carcinoma Bel-7402 cells, human colorectal adenocarcinoma HT-29 cells, and cervical adenocarcinoma HeLa cells^8,9^. However, the molecular mechanisms associated with its effects are not fully understood. In our preliminary experiments that screened for natural compounds that exhibit anti-angiogenic and apoptotic effects, RF was found to inhibit the proliferation of human umbilical vein endothelial cells (HUVECs) and exhibit cytotoxic effects. To our knowledge, the effect of RF on HUVECs has not yet been reported. Here, we investigated the anti-angiogenic and pro-apoptotic effects of RF on HUVECs. Moreover, the possible molecular mechanism involved in RF-induced apoptosis was elucidated.

## Results

### RF inhibited HUVEC proliferation and exhibited cytotoxicity

**Figure 1:**
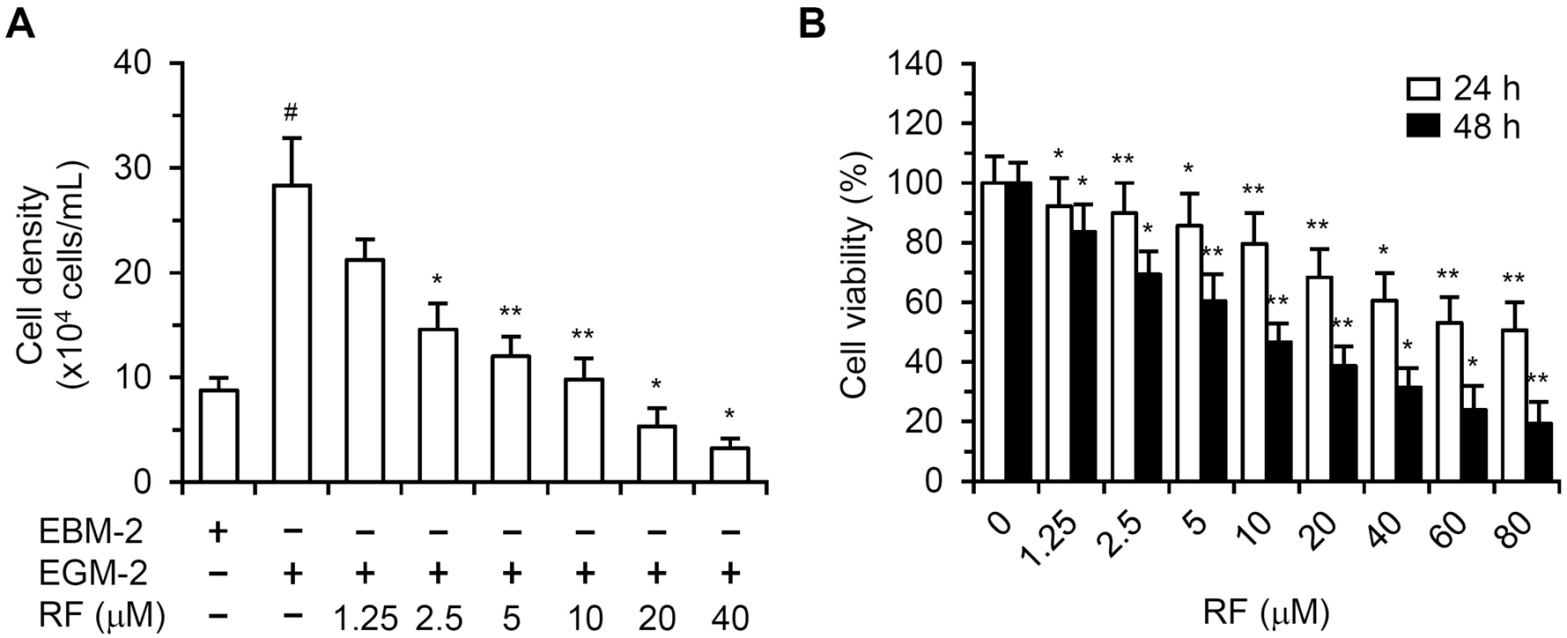
Analysis of HUVEC proliferation and RF cytotoxicity. (**A**) HUVECs were incubated for 48 h with EGM-2 containing different RF concentrations. The cells were trypsinized, stained with trypan blue, and counted using a hemocytometer. The cell density data are shown in the bar graph. The data are presented as the mean ± SD of three independent experiments; ^#^*p* < 0.05, compared to EBM-2-treated cells; *p < 0.05, **p < 0.01, compared to EGM-2-treated cells. (**B**) HUVECs were incubated for 24 or 48 h with EBM-2 containing different RF concentrations, and cell viability was assessed with the MTT assay. The data are presented as the mean ± SD of three independent experiments; *p < 0.05, **p < 0.01, compared to the control.

To determine the effect of RF on HUVEC proliferation, cells were incubated for 48 h in EGM-2 containing different RF concentrations. The viable cell density was measured after trypsinization and trypan blue staining. EGM-2 increased HUVEC proliferation by 223 (± 51.7)% (Fig. 1A). The presence of 1.25, 2.5, 5, or 10 μM of RF decreased HUVEC proliferation by 36.2 (± 10.1), 70.2 (± 12.7), 83.3 (± 9.7), or 94.5 (± 10.2)%, respectively. The density of HUVECs treated with 20 or 40 μM RF was lower than that of HUVECs cultured in EBM-2. To evaluate RF cytotoxicity, HUVECs were incubated with EBM-2 containing different RF concentrations for 24 or 48 h, and viability was measured by the MTT assay. Treatment with 1.25, 2.5, 5, 10, 20, 40, 60, or 80 μM RF for 24 h decreased the viability of HUVECs by 7.8 (± 7.2), 10.2 (± 9.5), 14.4 (± 10.2), 20.4 (± 10.9), 31.6 (± 9.5), 39.4 (± 9.2), 46.8 (± 8.9), or 49.4 (± 9.4)%, respectively (Fig. 1B). HUVEC viability decreased by 16.5 (± 8.1), 30.7 (± 7.3), 39.5 (± 7.9), 53.4 (± 6.5), 61.4 (± 6.8), 68.4 (± 6.6), 76.2 (± 7.2), or 80.7 (± 6.9)%, respectively, after treatment with 1.25, 2.5, 5, 10, 20, 40, 60, or 80 μM RF for 48 h. The 50% inhibitory concentration of 48-h RF treatment was 8.7 ± 0.6 μM.

### RF induced apoptosis in HUVECs

**Figure 2:**
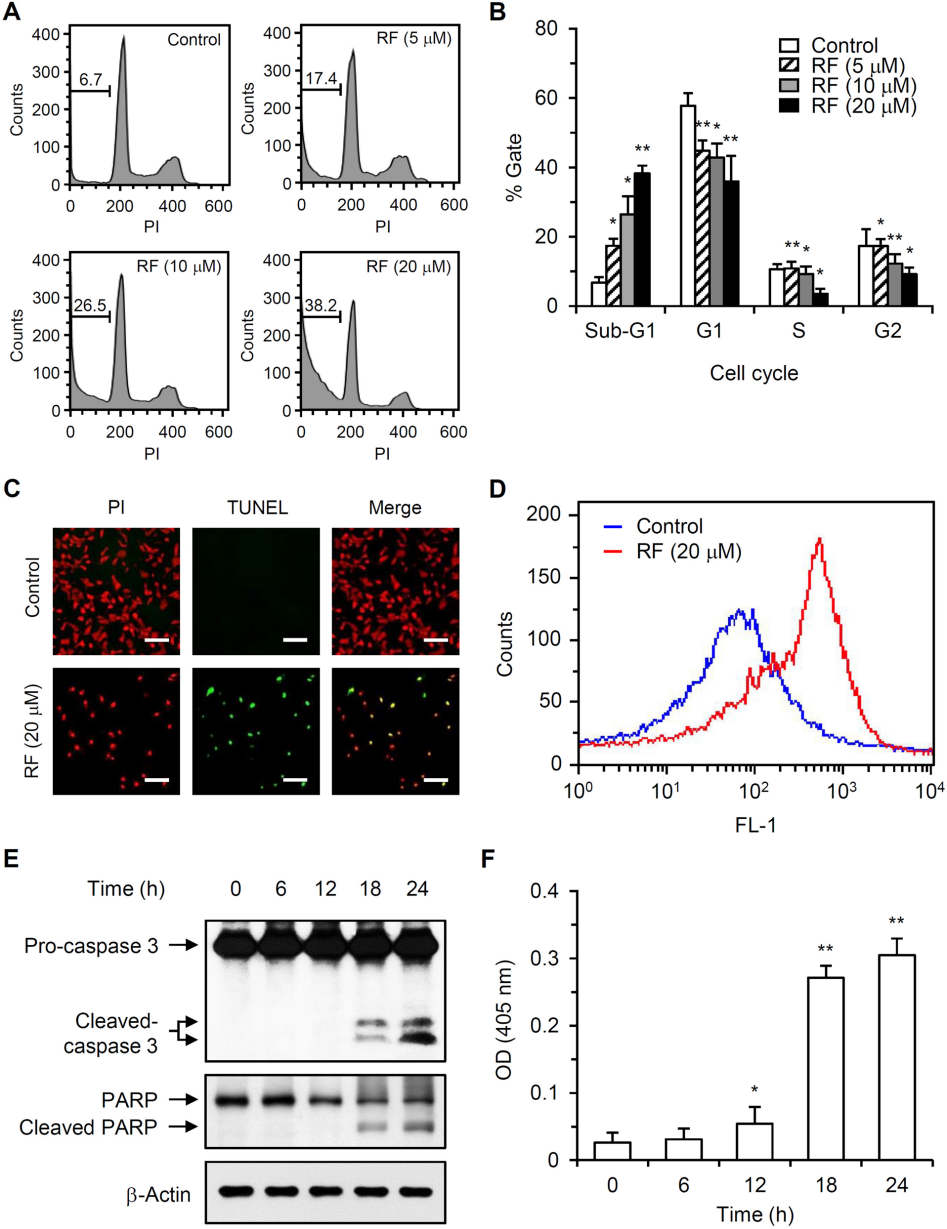
Sub-G1 cell populations, TUNEL staining, and analysis of caspase 3 activation in RF-treated HUVECs. (**A**) HUVECs were treated with different RF concentrations for 36 h, and the content of DNA was analyzed by flow cytometry after PI staining. (**B**) Data from three independent experiments of (**A**) are presented as the mean ± SD; *p < 0.05, **p < 0.01, compared to the control. (**C**) HUVECs were treated with 20 µM RF for 24 h, fixed, permeabilized, and stained with an enzyme buffer containing terminal deoxynucleotidyl transferase and fluorescein-dUTP. After PI staining, the cells were imaged at × 40 magnification (scale bar, 100 μm). (**D**) TUNEL-positive cells were also analyzed by flow cytometry. (**E**) HUVECs were treated with 20 μM RF, total protein was extracted, and pro-caspase 3, cleaved-caspase 3, PARP, and cleaved PARP levels were determined by western blotting. (**F**) Caspase 3 activity in the protein extracts was measured by colorimetric caspase 3-activity assays. The data are presented as the mean ± SD of three independent experiments; *p < 0.05, **p < 0.01, compared to the control.

To determine whether RF induced apoptosis, cell cycle analysis of sub-G1 cells was performed. The DNA content of HUVECs treated with 5, 10, or 20 μM RF for 36 h was analyzed by flow cytometry after PI staining. Treatment with 5, 10, or 20 μM RF increased the number of cells in the sub-G1 population by 159.7 (± 30.3), 295.5 (± 76.7), or 470 (± 34.1)%, respectively (Fig. 2A,B). Apoptosis induction by RF was further characterized by the TUNEL assay with a confocal microscope (Fig. 2C). The TUNEL assays showed that RF-treated cells were fluorescein-labeled, whereas control cells were not, which was further confirmed by flow cytometry (Fig. 2D). Caspase 3 and PARP activation was determined in 20 μM RF-treated HUVECs. RF treatment increased the level of cleaved caspase 3 (~ 17 and ~ 19 kDa) and induced PARP cleavage (Fig. 2E). Caspase 3 activity in RF-treated cell lysates was measured by a colorimetric caspase 3 enzyme assay (Fig. 2F). RF increased caspase 3 activity in cell lysates.

### RF increased the expression of Bad, Bax, and phosphorylated p53

**Figure 3:**
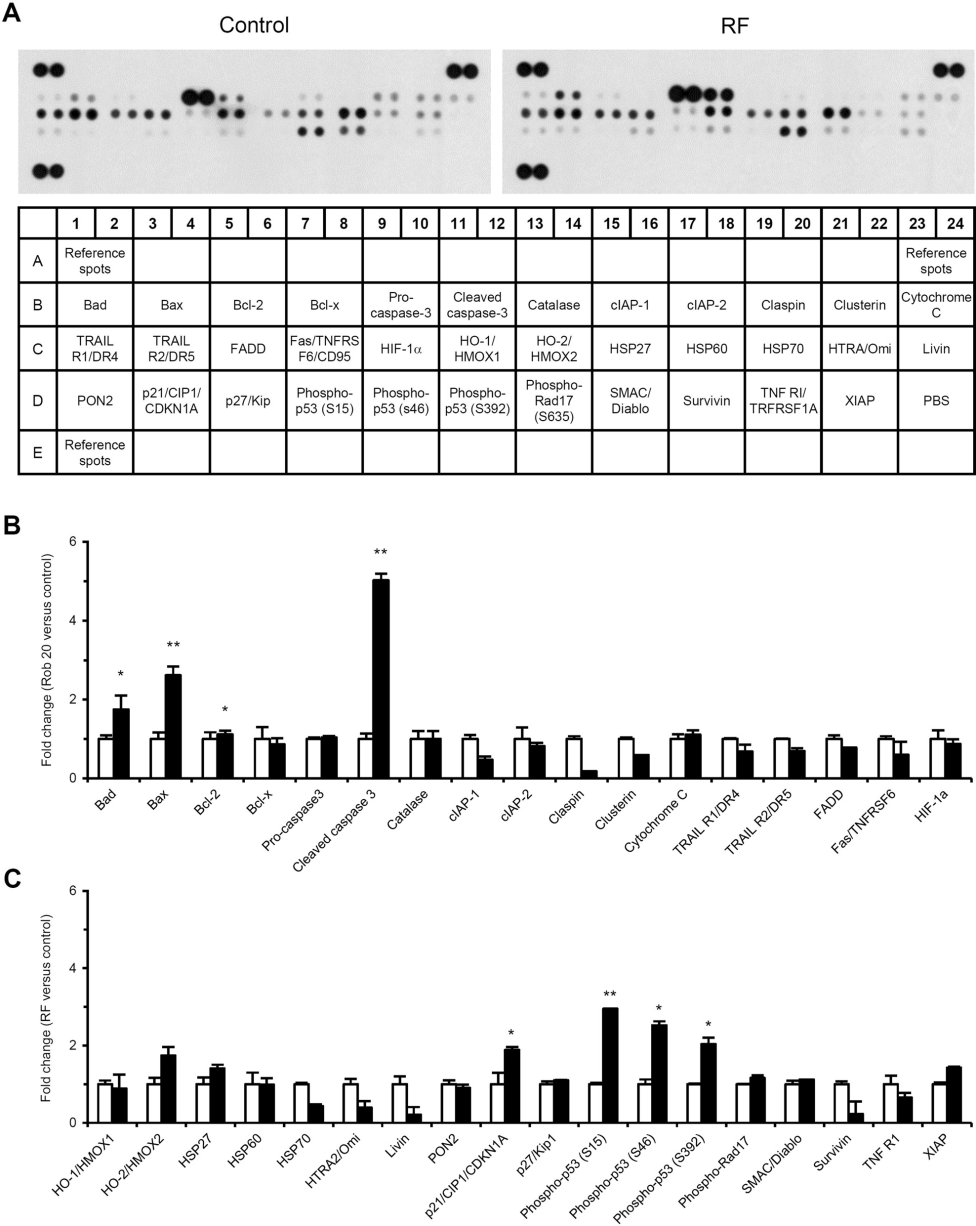
Effects of RF on apoptosis-related protein expression. (**A**) HUVECs were treated with 20 μM RF for 36 h, protein extracts were prepared, and apoptosis-associated protein-expression levels were determined. (**B**, **C**) Relative protein expression levels were estimated after quantifying the pixel densities in autoradiograms using ImageJ. Data from three independent experiments of (**A**) are presented as the mean ± SD; *p < 0.05, **p < 0.01, compared to the control.

To explore the potential molecular targets that mediate RF-induced apoptosis, we analyzed expression changes of apoptosis-associated proteins using a human apoptosis antibody array (Fig. 3). The levels of cleaved caspase 3, Bax, Bad, p21, and phosphorylated p53 (S15, S46, S392) were clearly increased in RF-treated cells compared to untreated control cells. RF treatment downregulated cIAP-1, claspin, HSP70, HTRA2, livin, and survivin expression.

### RF decreased the mitochondrial membrane potential and induced apoptosis via an intrinsic death pathway

**Figure 4:**
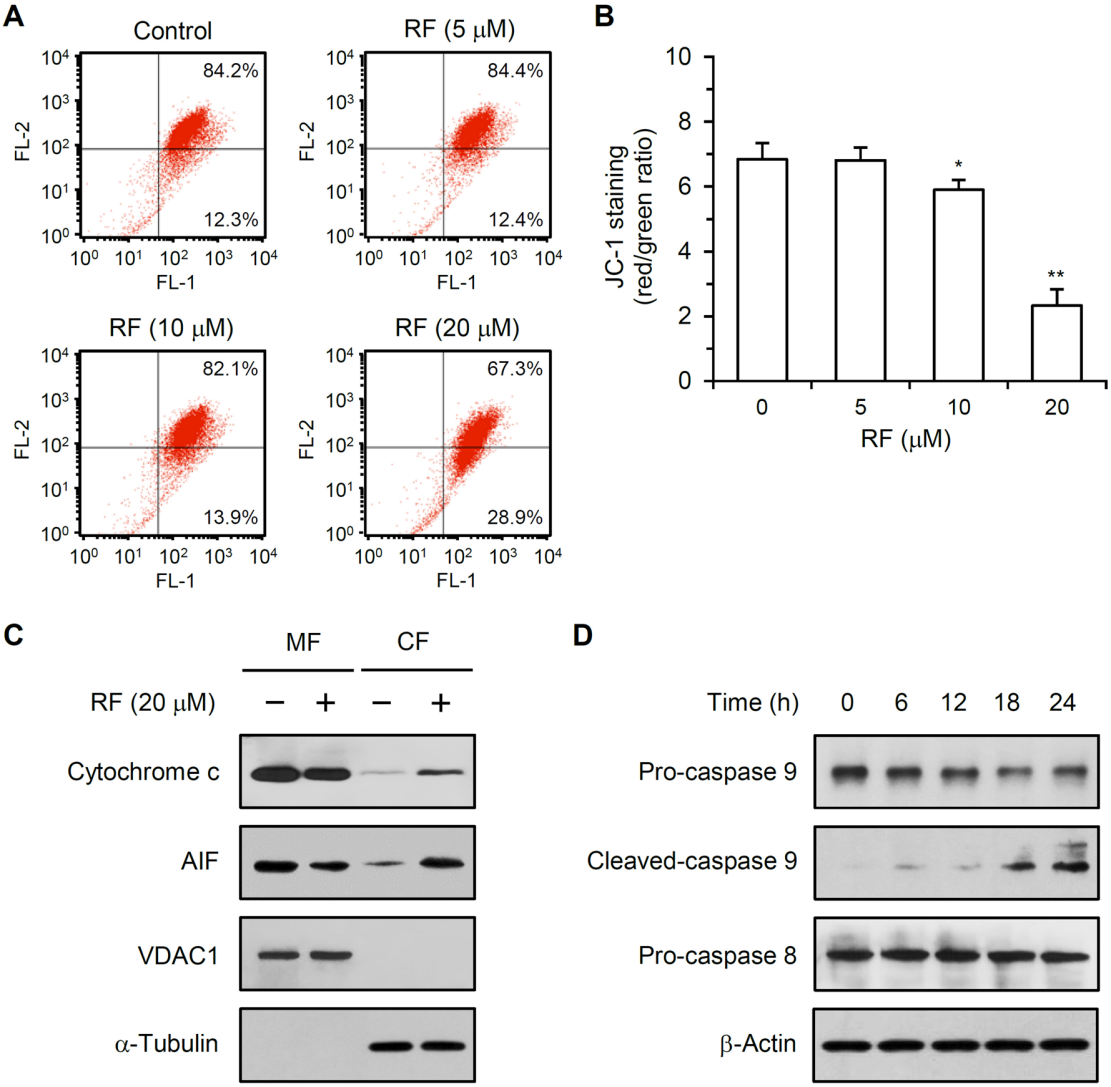
Effect of RF on the mitochondrial membrane potential and caspase 9 activation. (**A**) HUVECs were treated with different RF concentrations for 24 h, stained with JC-1, trypsinized, and resuspended in PBS. JC-1 fluorescence was measured at 530 nm (FL-1, green, monomeric form) and 590 nm (FL-2, red, aggregated form) by flow cytometry. (**B**) The ratio of aggregated JC-1 to monomeric JC-1 is shown. The data are presented as the mean ± SD of three independent experiments; *p < 0.05, **p < 0.01, compared to the control. (**C**) Mitochondrial fraction and cytoplasmic fraction (MF and CF) were collected from 20 μM RF-treated HUVECs. Equal amounts of each fraction were separated by SDS-PAGE, and cytochrome c and AIF levels were detected by western blotting. VDAC1 and α-tubulin were detected as controls to ensure that the MF and CF were not cross-contaminated. (**D**) HUVECs were treated with 20 μM RF, total protein was extracted, and pro-caspase 9, cleaved-caspase 9, pro-caspase 8, and cleaved-caspase 8 levels were determined by western blotting.

HUVECs were treated with 5, 10, or 20 μM RF for 24 h and stained with a fluorescent mitochondria-specific cationic dye (JC-1). The fluorescence intensities of the JC-1 monomer (530 nm) and aggregate (590 nm) were measured by flow cytometry. RF treatment increased the green fluorescence intensity of JC-1 (Fig. 4A). RF treatment (10 or 20 μM) for 24 h decreased the ratio of the aggregated form of JC-1 to the monomeric form of JC-1 by 13.9 (± 4.4) or 65.9 (± 7.3)%, respectively, compared to that in untreated control cells (Fig. 4B).

Mitochondrial and cytoplasmic protein fractions were prepared from 20 μM RF-treated HUVECs and control cells. RF treatment increased cytoplasmic cytochrome c and AIF levels (Fig. 4C). VDAC1 and α-tubulin levels were determined to ensure that the mitochondrial and cytoplasmic fractions were not cross-contaminated. RF treatment decreased pro-caspase 9 levels and increased cleaved caspase 9 levels (Fig. 4D). However, pro-caspase 8 levels were not affected by RF treatment. Cleaved caspase 8 was not detected (data not shown).

### RF induced cell cycle arrest at G0/G1 phase

**Figure 5:**
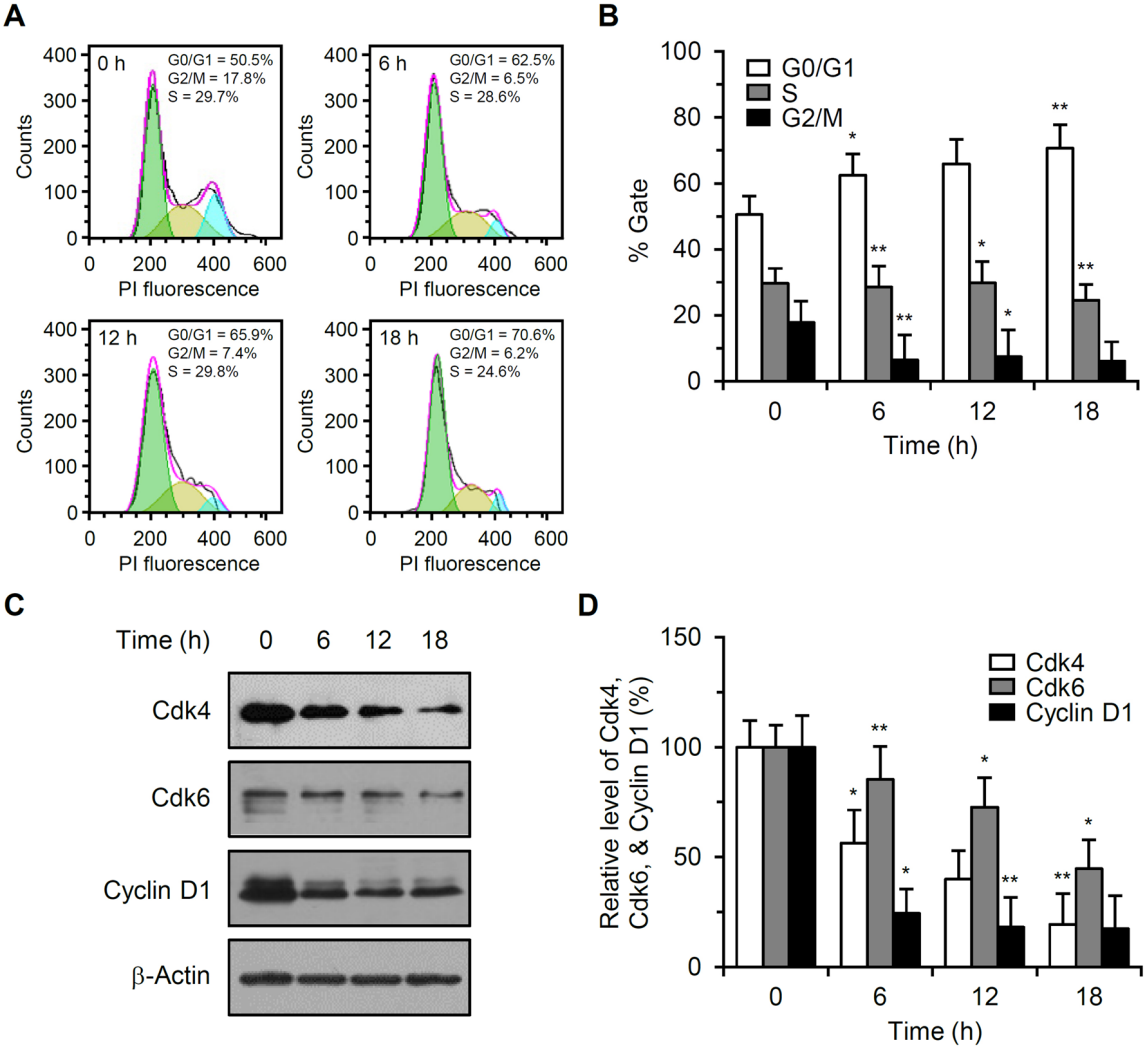
Effect of RF on cell cycle progression in HUVECs. (**A**) HUVECs were treated with 20 μM RF, viable cells were collected by trypsinization, and DNA content was analyzed after PI staining. (**B**) The percentages of cells in G0/G1, S, and G2/M phase in three independent experiments (**A**) are expressed as the mean ± SD; *p < 0.05, **p < 0.01, compared to the control. (**C**) Protein extracts were prepared from cells treated with 20 μM RF, and Cdk4, Cdk6, and cyclin D1 levels were determined by western blotting. (**D**) Cdk4, Cdk6, and cyclin D1 levels were measured by ImageJ. Data from three independent experiments of (**C**) are presented as the mean ± SD; *p < 0.05, **p < 0.01, compared to the control. Cdk4, Cdk6, and cyclin D1 levels in RF-untreated control cells were set to 100%.

HUVECs were treated with 20 μM RF, viable cells were collected by trypsinization, and cell cycle analysis was performed after PI staining. The percentage of control cells in G0/G1 phase was 50.5 (± 5.7)%. Treatment with 20 μM RF for 6, 12, or 18 h increased this percentage to 62.5 (± 6.4), 65.9 (± 7.5), or 70.6 (± 7.2)%, respectively (Fig. 5A,B). RF treatment decreased the percentage of cells in G2/M phase. Total protein extracts were also prepared from viable cells and western blot analysis was performed. RF treatment reduced Cdk4, Cdk6, and cyclin D1 expression (Fig. 5C,D).

### RF inhibited CT-26 allograft tumor growth and angiogenesis in a mouse model of colon carcinoma

**Figure 6:**
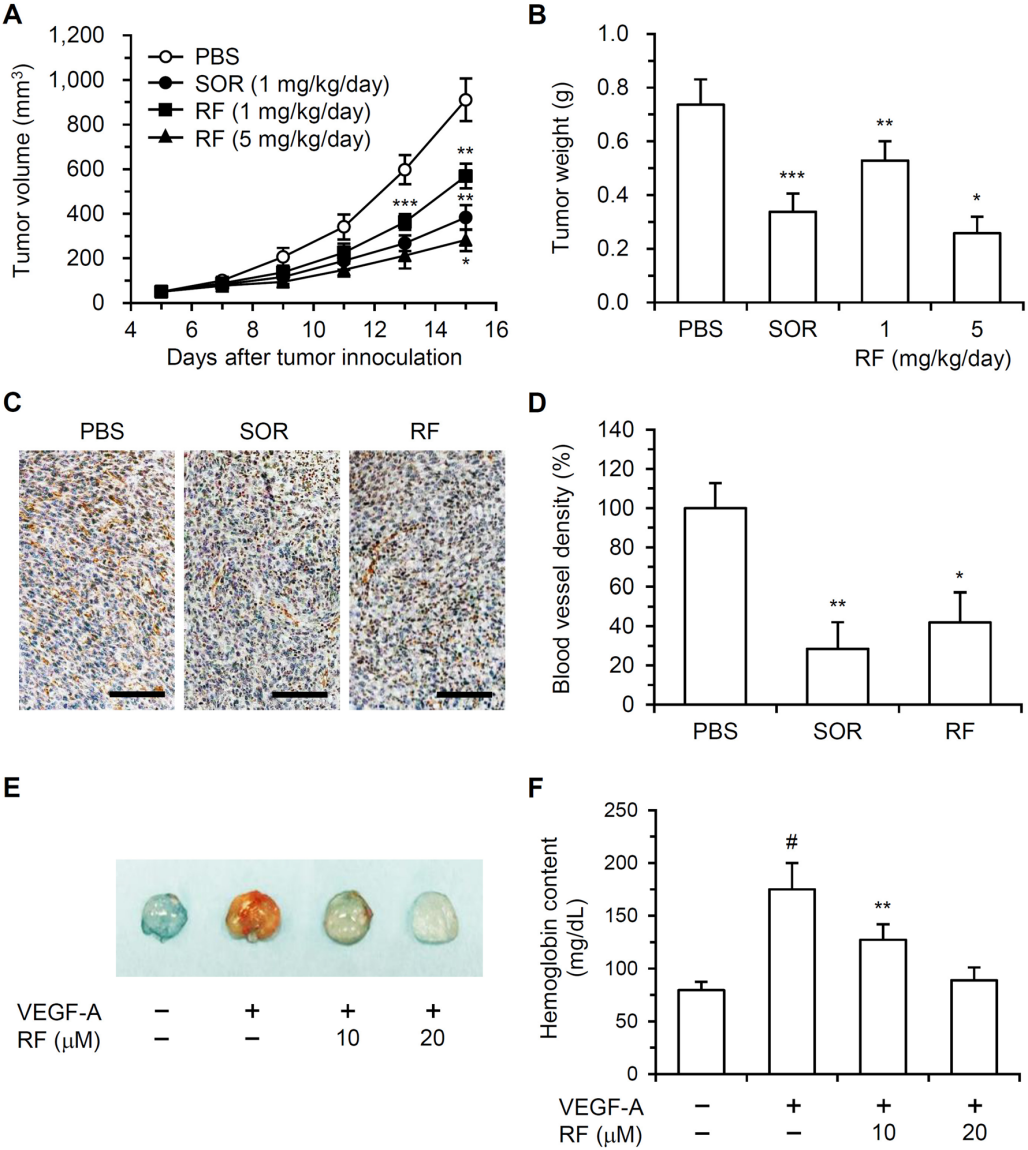
Effect of RF on tumor growth and tumor-induced angiogenesis in an animal CT-26 allograft colon carcinoma model and capillary vessel formation in an in vivo Matrigel plug. (**A**) CT-26 cells (5 × 10^5^ in 200 μL PBS) were subcutaneously injected into the right flank of BALB/c mice. After the establishment of tumors (5 days; ~ 50 mm^3^), the mice were injected peritumorally with RF (1 or 5 mg/kg/day in PBS with 0.2% DMSO), SOR (1 mg/kg/day in PBS with 0.2% DMSO), or PBS with 0.2% DMSO. The tumor volumes are presented as the mean ± SE; *p < 0.05, **p < 0.01, ***p < 0.001, compared to the PBS-treated control group. (**B**) Tumor weight after necropsy is presented as the mean ± SE; *p < 0.05, **p < 0.01, ***p < 0.001, compared to the PBS-treated control group. (**C**) Blood vessel density in tumor sections was determined using immunohistochemical analysis of CD31. All tumor sections were digitized, and microscopy images were captured at 100 × magnification (scale bar, 100 µm). (**D**) The density of immunohistochemical staining for CD31 was analyzed using ImageJ and is presented in the bar graph. The intensity of immunohistochemical staining for CD31 in the PBS-treated control group was set to 100%. The data are presented as the mean ± SD; *p < 0.05, **p < 0.01, compared to the PBS-treated control. (**E**) Matrigel containing VEGF-A (500 ng/mL) and/or RF (10, 20 μM) was injected bilaterally into the flank areas of BALB/c mice. At seven days post-injection, the Matrigel plugs were excised and imaged. (**F**) The hemoglobin content in the Matrigel plugs was determined by a colorimetric hemoglobin assay. The data are presented as the mean ± SD of three independent experiments; ^#^*p* < 0.05, compared to the control group not treated with VEGF-A or RF; **p < 0.01, compared to the VEGF-A-treated group.

The anti-tumor activity of RF was investigated in a mouse model of CT-26 cell-derived colon carcinoma. Sorafenib (SOR) was used as a positive control. In control mice (treated with PBS/0.2% dimethyl sulfoxide [DMSO]), tumors grew rapidly and reached an average volume of 910.8 ± 95.1 mm^3^ on day 15 after inoculation with CT-26 cells (Fig. 6A). In mice treated with 1 or 5 mg/kg/day RF, primary-tumor size (568.7 ± 55.0 or 282.1 ± 44.8 mm^3^) decreased by 37.5 (± 6.1) or 69.0 (± 5.4)%, respectively, compared to that in the PBS-treated control group. The average tumor volume of mice treated with SOR (1 mg/kg/day) was 382.9 ± 60.2 mm^3^. Tumor weight in the 1 or 5 mg/kg/day RF-treated group decreased by 28.3 (± 9.6) or 65.1 (± 8.2)%, respectively, compared to that in the PBS-treated control group (Fig. 6B). SOR reduced tumor weight by 54.2 (± 8.8)% compared to that in the PBS-treated control group. To estimate the anti-angiogenic effect of RF, immunohistochemical analysis for CD31 was performed on CT-26-derived tumor specimens. RF reduced the density of blood vessels stained with anti-CD31 (Fig. 6C). RF and SOR treatment (1 mg/kg/day) decreased blood vessel density by 58.2 (± 15.2) and 70.4 (± 13.5)%, respectively, compared to that in PBS-treated control group (Fig. 6D).

### RF suppressed blood vessel formation in in vivo Matrigel plugs

To explore the inhibitory effect of RF on angiogenesis in vivo, a Matrigel plug assay was performed in female BALB/c mice. VEGF-A induced red capillary vessel formation within the Matrigels (Fig. 6E). RF significantly inhibited VEGF-A-stimulated capillary vessel formation. The hemoglobin content in VEGF-A-treated Matrigels increased by 120 (± 31.5)% compared to that in the VEGF-A-nontreated controls (Fig. 6D). The presence of 10 or 20 μM RF decreased the hemoglobin content in Matrigels by 50.1 (± 26.2) or 90.2 (± 25.1)%, respectively.

## Discussion

Angiogenesis involves extracellular matrix degradation, endothelial cell proliferation and migration, and new blood vessel formation with coordinated endothelial cells^13^. These processes are potential targets for novel anti-angiogenic agents. Endothelial cell apoptosis inhibits angiogenesis^14^. Therefore, identifying novel anti-angiogenic agents capable of inhibiting endothelial cell proliferation or inducing endothelial cell apoptosis in the tumor layer is useful for treating tumors and angiogenesis-related diseases. RF may exhibit anti-tumor activity and reduce human carcinoma cell viability^8,9^. However, studies on the anti-angiogenic and/or pro-apoptotic effects of RF have not been reported. In vitro inhibitory activities that reduce endothelial cell proliferation, migration, and tube formation are important determinants of anti-angiogenic effects. RF exhibited anti-angiogenic effects, inhibiting the proliferation (Fig. 1A), migration, and tube formation of HUVECs (Supplementary Fig. S1). RF inhibited HUVEC viability through its cytotoxic activity (Fig. 1B). These results indicate that the anti-angiogenic effects of RF were caused by cytotoxicity. The effect of RF was also evaluated using human lymphatic microvascular endothelial cells (HLMECs). RF reduced the proliferation of HLMECs and showed cytotoxicity that inhibited HLMEC viability, similar to the results obtained with HUVECs (Supplementary Fig. S2).

Both cytotoxic chemotherapy and anti-angiogenic therapy depend on endothelial cell apoptosis^15,16^. Endothelial cell apoptosis in the vascular tumor bed precedes tumor cell apoptosis. RF induced HUVEC apoptosis, as determined by the detection of sub-G1 cell populations and TUNEL assays (Fig. 2A–D). Apoptosis can be classified as caspase-dependent or caspase-independent^17^. Caspases are cysteine proteases that mediate apoptosis and are essential for initiating and executing cell death. RF induced caspase 3 and PARP activation (Fig. 2E,F), suggesting that RF-induced apoptosis involves a caspase-dependent apoptotic mechanism. Caspase 3 is an effector caspase responsible for the morphological and biological changes seen in apoptotic cells. PARP proteins are activated by caspase signaling and participate in DNA repair, genome stability, and apoptosis^18^. Caspase-dependent apoptosis can be initiated through the intrinsic or extrinsic pathways^17^. The intrinsic pathway is mediated by mitochondria and can be induced by hypoxia, radiation, viral infections, and DNA damage. The Bcl-2 protein family is important for the intrinsic apoptosis pathway and comprises pro-apoptotic members (Bax, Bad, Bid, and Bak) and anti-apoptotic members (Bcl-2, Bcl-xL, and Mcl-1)^19^. The ratio of pro-apoptotic to anti-apoptotic members (Bax/Bcl-2) is a key factor that regulates apoptosis. Changes in the Bax/Bcl-2 ratio can affect mitochondrial membrane permeability and the release of specific pro-apoptotic proteins such as cytochrome c and AIF. We found that RF increased Bax and Bad expression, without affecting Bcl-2 or Bcl-xL expression (Fig. 3), thereby increasing the Bax/Bcl-2 ratio in HUVECs. RF decreased the mitochondrial membrane potential and caused mitochondrial cytochrome c and AIF release (Fig. 4A–C). RF also induced caspase 9 activation (Fig. 4D). The extrinsic apoptosis pathway is a relatively fast cell death process mediated by activated caspase 8 and death receptors such as tumor necrosis factor receptor, Fas, death receptor (DR) 3, DR 4, and DR 5^17^. RF did not affect DR expression levels (Fig. 3) or caspase 8 activation (Fig. 4D), indicating that its effect was independent of the death receptor-mediated extrinsic pathway. Collectively, our results indicate that RF induced apoptosis in HUVECs via the mitochondria-mediated intrinsic pathway; RF increased the Bax/Bcl-2 ratio, induced mitochondrial cytochrome c and AIF release, and activated caspase 9 and caspase 3, which in turn activated PARP. A reduction in mitochondrial membrane potential can be associated with increased reactive oxygen species (ROS)^20^. The relationship between RF and ROS is poorly understood. Extracts of *Selaginella* species (which contain RF and having anti-tumor activity) have been used to treat sore throats, rheumatoid arthritis, and some cancers^21^. *Selaginella doederleinii* extract induces apoptosis in human nasopharyngeal cancer, which is mediated by ROS-mediated mitochondrial dysfunction^22^. Collectively, these results suggest that RF-induced apoptosis may be associated with ROS production and thereby activate the mitochondria-mediated intrinsic pathway.

P53 is highly induced following various stress signals such as DNA damage, oncogene activation, and nutrient deprivation^23^. Cell cycle arrest and apoptosis are the most prominent outcomes of p53 activation and are regulated by the degree of cellular stress. Phosphorylation is a major modification that enhances transcription transactivation by p53. RF activated p53 by enhancing phosphorylations at S15, S46, and S392 (Fig. 3). Phosphorylation at S15 and S46 following DNA damage can induce p53-mediated cell cycle arrest and apoptosis^24^. S392 phosphorylation is a common and integral event during the induction of p53 by diverse stressors^25^. Cell cycle arrest by p53 is primarily mediated by the induction of p21 transcription^26^. P21 binds to the cyclin D1-Cdk4/6 complex, resulting in G0/G1 arrest. RF increased p21 expression (Fig. 3), although cell cycle arrest was not observed in cells treated with 20 μM RF for 36 h (Fig. 2). This discrepancy might have occurred because most cells underwent severe cell death under the experimental conditions. To analyze RF-dependent cell cycle arrest, only viable cells were collected after RF treatment and cell cycle was analyzed by flow cytometry. RF induced cell cycle arrest at G0/G1 phase (Fig. 5A,B). Accumulation of cells at Sub-G1 population was not observed. This might be because only viable cells were used for cell cycle analysis. RF also downregulated cyclin D1, Cdk4, and Cdk6 expression (Fig. 5C,D).

Furthermore, the in vivo anti-angiogenic effects of RF were determined using an animal CT-26 colon carcinoma model and in vivo Matrigel plug assays in BALB/c mice. RF reduced the tumor size and weight (Fig. 6A,B). The blood vessel density in RF-treated tumors decreased significantly. The anti-tumor and anti-angiogenic effects of RF were less robust than the effects of SOR, an anti-angiogenic agent that inhibits tumor growth and angiogenesis^27^. RF inhibited capillary vessel formation within VEGF-A-stimulated Matrigel plugs (Fig. 6C). Hemoglobin content also decreased in RF-treated Matrigel plugs (Fig. 6F). These results indicate that RF exhibited anti-angiogenic activity in vivo. To our knowledge, this is the first study demonstrating that RF can inhibit angiogenesis in vivo.

In conclusion, RF inhibited HUVEC viability and induced cell cycle arrest (G0/G1 phase) and apoptosis via the mitochondria-mediated intrinsic pathway. In addition, RF suppressed new blood vessel formation in vivo. To our knowledge, this is the first report demonstrating that RF induces apoptosis in HUVECs and shows anti-angiogenic effects in vivo. Our findings suggest that RF serves as an anti-angiogenic agent for inhibiting angiogenesis-stimulated tumor growth and metastasis.

## Materials and methods

### Materials

RF (> 98% pure) was purchased from Chem-Norm Biotech. Co., Ltd. (Wuhan, China). SOR was purchased from Sigma-Aldrich (St. Louis, MO, USA). RF and SOR were dissolved in DMSO. 0.1% (v/v) DMSO was used as a control in all experiments. An Endothelial Growth Medium-2 Bullet Kit (EGM-2) and Endothelial Basal Medium-2 (EBM-2) were purchased from Lonza (Basel, Switzerland). Fetal bovine serum (FBS) was purchased from HyClone (Logan, UT, USA). Gelatin, 3-[4,5-dimethylthiazol-2-yl]-2,5-diphenyltetrazolium bromide (MTT), Harris hematoxylin solution, propidium iodide (PI), and horseradish peroxidase (HRP)-conjugated secondary antibodies (anti-mouse and anti-rabbit IgG) were purchased from Sigma-Aldrich. Matrigel basement membrane matrix was purchased from Corning (Corning, NY, USA). 5,5ʹ,6,6ʹ-Tetrachloro-1,1ʹ3,3ʹ-tetraethyl-benzimidazoly-carbocyanine iodide (JC-1), radioimmunoprecipitation assay (RIPA) buffer, SuperSignal™ West Pico and Femto chemiluminescent substrate were purchased from Thermo Fisher Scientific, Inc. (Waltham, MA, USA). A protease inhibitor cocktail was purchased from Roche (Indianapolis, IN, USA). Vascular endothelial growth factor A (VEGF-A) and a CD31 antibody were purchased from Abcam (Cambridge, MA, USA). Primary antibodies against caspase 3, poly (ADP-ribose) polymerase (PARP), procaspase 9, caspase 9, cyclin-dependent kinase 4 (Cdk4), Cdk6, cyclin D1, and β-actin were purchased from Cell Signaling Technology, Inc. (Danvers, MA, USA). Antibodies against apoptosis-inducing factor (AIF), voltage-dependent anion-selective channel-1 (VDAC1), and α-tubulin were purchased from Santa Cruz Biotechnology, Inc. (Dallas, TX, USA). A cytochrome c antibody was purchased from BD Biosciences (San Jose, CA, USA).

### Cell culture

Primary HUVECs were obtained from Lonza and maintained in EGM-2 (EBM-2 supplemented with 2% FBS, 0.4% human fibroblast growth factor-2, 0.1% vascular endothelial growth factor, 0.1% arginine 3 insulin-like growth factor-1, 0.1% human epidermal growth factor, 0.04% hydrocortisone, 0.1% ascorbic acid, 0.1% heparin, and 0.1% GA-1000) in a humidified incubator with 5% CO_2_ at 37 °C. HUVECs were cultured using 0.1% gelatin-coated well plates or cell culture dishes. HUVECs at passage 5 or 6 were used in all experiments.

### HUVEC proliferation assay

HUVECs were seeded in 24-well plates (2 × 10^4^ cells/well). After incubation overnight, the cells were treated with EGM-2 containing different RF concentrations and incubated for an additional 48 h. The cells were trypsinized and stained with trypan blue, and the viable cells were counted using a hemocytometer.

### Cytotoxicity assay

The cytotoxicity of RF was measured using an MTT colorimetric assay. Briefly, HUVECs were seeded in 96-well plates (1 × 10^4^ cells/well). After cultivation overnight, the cells were treated with EBM-2 containing different RF concentrations and further incubated for 24 or 48 h. MTT solution (20 μL; 5 mg/mL in phosphate-buffered saline [PBS]) was added to each well, and the cells were incubated at 37 °C for 4 h. After the medium was removed, DMSO (100 μL) was added to dissolve the formazan. The absorbance of the samples was measured at 550 nm using a microplate reader (BioTek, Winooski, VT, USA). Cell viability was calculated as follows: cell viability (%) = ([OD_RF_ – OD_Blank_]/[OD_Control_ – OD_Blank_]) × 100.

### Cell cycle analysis

HUVECs were seeded in 100-mm cell culture dishes (3 × 10^5^ cells/dish), incubated for 48 h, and treated with EGM-2 containing different RF concentrations for 36 h. The cells were fixed, stained with PI, and subjected to cell cycle analysis as described previously^28^.

### Terminal deoxynucleotidyl transferase-mediated dUTP nick-end labeling (TUNEL) assay

HUVECs were seeded in confocal dishes (SPL Life Sciences, Pocheon-si, Korea; 3 × 10^5^ cells/dish). After incubation overnight, the cells were treated with EGM-2 with or without 20 μM RF for 24 h and collected by trypsinization. TUNEL assays were performed using an In Situ Cell Death Detection Kit (Roche). For fluorescence-microscopy analysis, the cells were mounted onto glass slides and examined under a confocal laser-scanning microscope (FV1000; Olympus, Waltham, MA, USA). For flow cytometry analysis, the cells were resuspended in 1 mL PBS. Green fluorescence (TUNEL-positive cells) was measured using a 530 ± 30 nm bandpass filter.

### Caspase 3 activity assay

Caspase 3 activity was assessed using a Colorimetric Caspase 3 Enzyme Assay Kit (Abcam). Briefly, RF-treated HUVECs were resuspended in lysis buffer and incubated on ice for 30 min. After centrifugation at 14,000 × *g* for 10 min at 4 °C, protein extracts were collected and quantified using a DC Protein Array Kit (Bio-Rad, Hercules, CA, USA). Protein extracts (500 µg) were incubated with reaction buffer containing 2 mM Ac‐DEVD‐AFC and 10 mM DTT at 37 °C for 30 min. Caspase activity was determined by measuring the absorbance at 405 nm.

### Human apoptosis antibody array

The expression levels of apoptosis-related proteins were determined using a Human Apoptosis Array Kit (R&D Systems, Minneapolis, MN, USA). Nitrocellulose-membrane sheets containing duplicate spots of 35 apoptosis-related proteins were incubated with blocking buffer at room temperature for 1 h. Total-protein lysates were extracted from HUVECs treated with 20 μM RF or 0.1% DMSO (as a control). Protein lysates (50 µg) were incubated overnight with the membrane sheets at 4 °C. After rinsing, the sheets were incubated with biotin-conjugated antibodies at room temperature for 1 h and probed with HRP-conjugated streptavidin for 30 min. The membrane sheets were then treated with a chemi reagent mixture (provided by the kit) and exposed to X-ray film. Relative protein levels were estimated by comparing the pixel densities of protein spots and quantified using ImageJ.

### Mitochondrial membrane potential analysis

HUVECs incubated for 24 h in EGM-2 containing 20 μM RF or 0.1% DMSO were treated with 10 μg/mL JC-1 cationic dye for 30 min at 37 °C in the dark and then trypsinized. The JC-1 fluorescence intensities (10,000 cells/sample) were measured by flow cytometry using FACSCalibur and CellQuest (BD Biosciences). Green and red fluorescence were detected at excitation/emission wavelengths of 485/530 and 550/590 nm, respectively, and analyzed with FlowJo v10 software (Tree Star Inc., Ashland, OR, USA).

### Preparation of mitochondrial and cytoplasmic fractions

HUVECs were incubated with EGM-2 containing 20 μM RF or 0.1% DMSO at 37 °C for 24 h. Mitochondrial and cytosolic fractions were prepared using a Mitochondria Isolation Kit for Cultured Cells (Thermo Fisher Scientific, Inc.). Protein concentrations in mitochondrial and cytoplasmic fractions were determined using a DC Protein Array Kit. Equal amounts of proteins were separated by sodium dodecyl sulfate–polyacrylamide gel electrophoresis (SDS-PAGE) and analyzed by western blotting.

### Western blot analysis

RF- or DMSO-treated HUVECS were lysed using RIPA buffer supplemented with protease inhibitor cocktail. After centrifugation at 14,000 × *g* for 20 min at 4 °C, protein extracts were collected and quantified. Protein samples were separated by SDS-PAGE and transferred to polyvinylidene fluoride membranes (Pall Corporation, Port Washington, NY, USA). The membranes were pre-incubated in blocking solution (5% [w/v] skim milk in tris-buffered saline [TBS] containing 0.1% [v/v] Tween 20) for 1 h and then incubated overnight with primary antibodies at 4 °C. All primary antibodies were diluted 1:2,000 in blocking solution or bovine serum albumin solution (3% [w/v] in TBS containing 0.1% Tween-20). The membranes were then probed with HRP-conjugated secondary antibodies (diluted 1:2,000 or 1:4,000 in blocking solution) at room temperature for 2 h. The protein bands were detected using either SuperSignal™ West Pico or Femto chemiluminescent substrate.

### Tumor growth in mice with CT-26 allografts

The animal care facility and experimental protocols were approved by the Kyung Hee University Institutional Animal Care and Use Committee (KHUASP[GC]-18–27). All animal care and experimental procedures were performed according to the Kyung Hee University guidelines for the care and use of laboratory animals. Five-week-old female BALB/c mice were purchased from Nara Biotech (Seoul, Korea). The mice were provided water and food ad libitum, and quarantined in a specific-pathogen-free environment with a 12-h light and 12-h dark photoperiod. To establish an animal allograft colon carcinoma model, 5 × 10^5^ CT-26 cells in 200 μL PBS were injected into the right flank areas of BALB/c mice. The tumors grew for 5 days to form visible masses. RF (1 or 5 mg/kg/day in PBS with 0.2% DMSO), SOR (1 mg/kg/day in PBS with 0.2% DMSO), or PBS (with 0.2% DMSO) was injected peritumorally into the mice for 10 days at two-day intervals. Tumor volumes and weights were measured as described previously^28^.

### Immunohistochemical analysis

Tumor specimens were immediately removed from sacrificed mice and prepared for immunohistochemical analysis. Tumors were fixed overnight in 10% neutral-buffered formalin, embedded in paraffin, and sectioned into 4-μm-thick slices. The tumor sections were deparaffinized by immersion in xylene, dehydrated in a graded series of ethanol, and washed with distilled water. Immunohistochemical analysis of CD31 was performed as described previously^28^.

### In vivo Matrigel-plug assay

Matrigel aliquots (0.4 mL) were mixed with RF (10 or 20 μM) or DMSO (0.1%, control) in the presence of VEGF-A (500 ng/ml). Matrigel mixtures were injected into the right flank areas of BALB/c mice. Seven days after injection, Matrigel plugs were excised and imaged using a digital camera. Hemoglobin content in Matrigel plugs was determined using a Hemoglobin Colorimetric Assay Kit (BioVision, Milpitas, CA, USA) and normalized based on Matrigel weight.

### Statistical analysis

All data are represented as the mean ± standard deviation (SD) or standard error (SE). Statistical comparisons were performed using one-way analysis of variance (ANOVA) followed by Student’s *t*-test using Microsoft Excel (Microsoft Corp., San Diego, CA, USA). *P* values less than 0.05 were considered statistically significant.

## Supplementary information

Supplementary file1 (PDF 485 kb)
