# Supplementary material for: Robustaflavone induces G0/G1 cell cycle arrest and apoptosis in human umbilical vein endothelial cells and exhibits anti-angiogenic effects in vivo
Source: Sci Rep. 2020 Jul 6;10:11070. doi: 10.1038/s41598-020-67993-5 (PMC7338547; doi:10.1038/s41598-020-67993-5)
Supplement: Supplementary file 1 — Supplementary file1 (PDF 485 kb) [file 41598_2020_67993_MOESM1_ESM.pdf]

Supplementary Information

Robustaflavone induces G0/G1 cell cycle arrest and apoptosis in human umbilical vein endothelial cells and exhibits anti-angiogenic effects *in vivo*

Woo Kyung Sim, Jong-Hwa Park, Ki-Young Kim & In Sik Chung*

Department of Genetic Engineering and Graduate School of Biotechnology, Kyung Hee University, Yongin 17104, Republic of Korea.

*Corresponding author: Department of Genetic Engineering and Graduate School of Biotechnology, Kyung Hee University, Yongin 17104, Republic of Korea. Tel: +82-31-201-2436; Fax: +82-31-202-9885; E-mail: ischung@khu.ac.kr.

Supplementary Fig. S1. Effect of RF on HUVEC migration and tube formation. (A) HUVECs in EBM-2 containing different RF concentrations were added to the upper chambers of transwell inserts with 8.0-μm pore polycarbonate membranes coated with 0.1% gelatin. EGM-2 was added to the lower chambers to induce cell migration. After an 18-h incubation, cells that migrated to the insert underside were fixed with methanol, stained with Harris hematoxylin solution, and imaged at ×40 magnification (scale bar, 100 μm). (B) Ten images/well were obtained, and HUVEC migration/320-mm^2 area was determined. The data are presented as the mean ± SD of three independent experiments; #$p < 0.05$, compared to EBM-2-treated cells; *$p < 0.05$, **$p < 0.01$, compared to EGM-2-treated cells. (C) Confluent HUVECs were scraped horizontally using a P200 pipette tip and treated for 16 h with EGM-2 containing different RF concentrations. The scraped fields were photographed at ×40 magnification (scale bar, 100 μm). (D) Distances between each wound edge are represented as the % migration. The migration of cells treated with EGM-2 alone was set to 100%. The data are presented as the mean ± SD of three independent experiments; *$p < 0.05$, **$p < 0.01$, compared to EGM-2-treated cells. (E) HUVECs in EGM-2 containing 1% FBS and different RF concentrations were added to Matrigel-precoated 48-well plates. After an 8-h incubation, the cells were imaged at ×20 magnification (scale bar, 200 μm). (F) Total HUVEC tube lengths were quantified using the ImageJ program. The data are presented as the mean ± SD of three independent experiments; #$p < 0.05$, compared to EBM-2-treated cells; *$p < 0.05$, **$p < 0.01$, compared to EGM-2-treated cells.

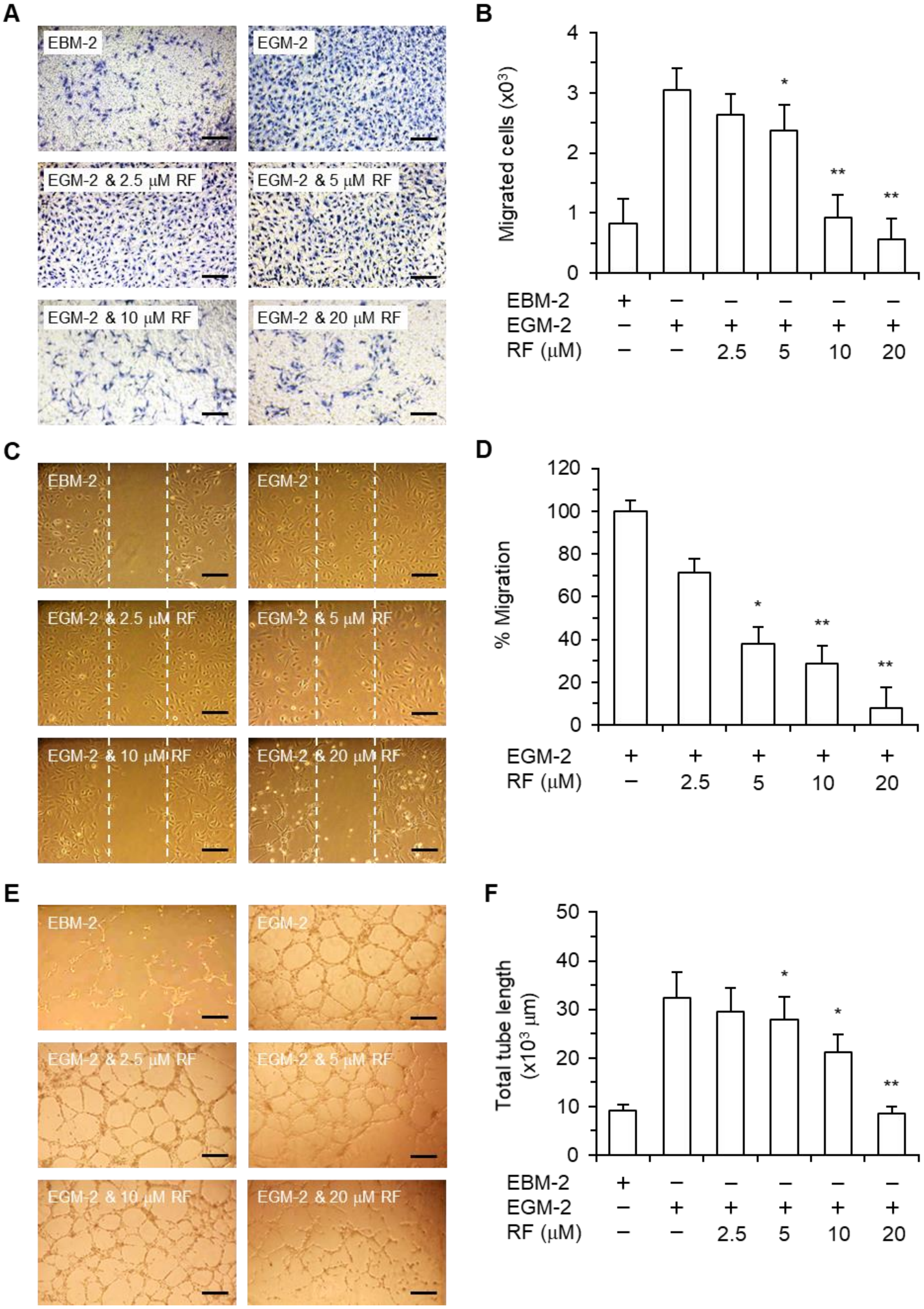
A
EBM-2
EGM-2
EGM-2 & 2.5 μM RF
EGM-2 & 5 μM RF
EGM-2 & 10 μM RF
EGM-2 & 20 μM RF
B
Migrated cells (x0³)
EBM-2
EGM-2
RF (μM)
2.5
5
10
20
C
D
% Migration
E
F
Total tube length (x10³ μm)

Supplementary Fig. S2. Effect of RF on the proliferation of human lymphatic microvascular endothelial cell (HLMEC) and RF cytotoxicity analysis. (A) HLMECs were obtained from Lonza and maintained in EGF-2MV (Lonza) supplemented with 10% FBS in a humidified incubator with 5% CO_2 at 37°C. HLMECs were incubated for 48 h with EGM-2MV containing different RF concentrations. The cells were trypsinized, stained with trypan blue, and counted using a hemocytometer. The cell density data are shown in the bar graph. The data are presented as the mean ± SD of three independent experiments; $^{\#}p < 0.05$, compared to EBM-2-treated cells; $^{*}p < 0.05$, $^{**}p < 0.01$, compared to EGM-2MV-treated cells. (B) HLMECs were incubated for 24 or 48 h with EBM-2 containing different RF concentrations, and cell viability was assessed with the MTT assay. The data are presented as the mean ± SD of three independent experiments; $^{*}p < 0.05$, $^{**}p < 0.01$, compared to the control.

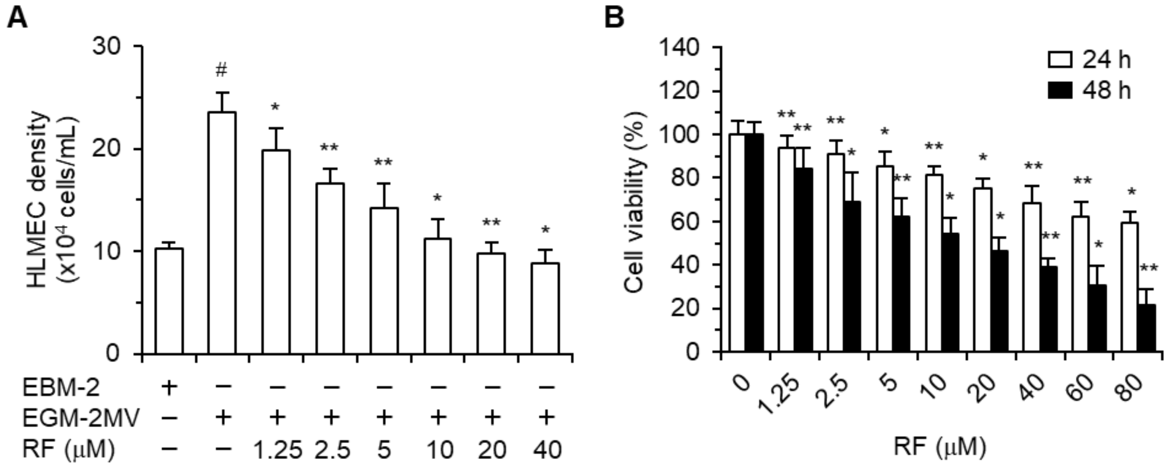
A
B
HLMEC density (x10^4 cells/mL)
30
20
10
0
#
*
**
EBM-2
EGM-2MV
RF (μM)
1.25
2.5
5
10
20
40
Cell viability (%)
140
120
100
80
60
40
24 h
48 h
0
60
80
RF (μM)
